# Stimulation of Estrus and Ovulation by Resynchronization in Kangal Sheep during Early Anestrus

**DOI:** 10.3390/vetsci10080499

**Published:** 2023-08-02

**Authors:** Abdurrahman Takci, Dursun Ali Dinc

**Affiliations:** 1 Department of Obstetrics and Gynecology, Faculty of Veterinary Medicine, Sivas Cumhuriyet University, Sivas 58140, Türkiye; 2 Department of Obstetrics and Gynecology, Faculty of Veterinary Medicine, Selcuk University, Konya 42130, Türkiye; dadinc@selcuk.edu.tr

**Keywords:** sheep, estrous stimulation, resynchronization, pregnancy

## Abstract

**Simple Summary:**

For the last 20 years, it has been aimed to prevent seasonality that limits fertility in sheep. Although numerous sexual stimulations were attempted to induce fertile ovulatory anestrus, desired rates of pregnancy could not be achieved through such one-time stimulations. It has been repeatedly proved that despite estrus being induced and sheep being mated, sheep were turned into deep anestrus if sheep were not conceived. Thus, herein a second chance of pregnancy was given to animals who could not conceive despite the activation of reproductivity. In this context, the method of resynchronization that has successfully been implemented over cows for years was tried for the first time in sheep reproduction following required modifications. The successful adaptation of these existing systematic applications to sheep will enable two consecutive progesterone-supported synchronizations. Thus, while progestative support is provided in the implantation phase to the ewes that conceive at the first stimulation, pregnancies will be increased by providing a second mating for those who do not become pregnant without losing time.

**Abstract:**

A total of 100 Kangal sheep were divided into four groups with the aim of investigating the effectiveness of resynchronization during anestrus for the first time in the literature. The groups were then divided into two further subgroups, namely the resynchronization subgroup group (hCG+resynch) and group (resynch)) and the no resynchronization subgroup (Group (hCG) and group (control)). All the groups started with progesterone-containing sponge insertion on Day 7. The sponge was removed after 7 days (on Day 0), and 600 IU eCG + 131.5 µg PGF2α was injected. The animals in group (hCG+resynch) and group (hCG) received hCG injection at the time of sponge administration. Accordingly, four different groups were established, i.e., resynchronization + hCG administration group (hCG+resynch); n:25), no resynchronization + hCG administration (group (hCG); n:25), resynchronization + no hCG administration (group (resynch); n:25), and no resynchronization + no hCG administration (Group (control); n:25). Estrus rates at the first application in group (hCG+resynch), group (hCG), group (resynch), and group (control) groups were 76%, 88%, 96%, and 76%, respectively, and pregnancy rates were 52%, 64%, 72%, and 60%, respectively; there were no intergroup statistical differences in the two parameters above. It was concluded that resynchronization performed with two consecutive stimulations during anestrus could help save time and provide a pregnancy rate at a level that can provide economic returns.

## 1. Introduction

There is an increased need for animal protein at present [1,2], and it has been suggested that climatic and environmental conditions will pose further challenges for cattle breeders across the world, especially in countries that supply the highest amounts of animal protein [3,4,5,6]. Therefore, the potential deficit in the supply of food of animal origin can be overcome by sheep breeding. Sheep breeding is a branch of animal husbandry where products of animal origin can be produced easily with fewer input costs through adequate reproductive management [7,8]. Notwithstanding the above, the fact that there is no off-season reproduction in sheep and that there is a long period of anestrus following birth is associated with a seasonal fluctuation in the product supply (milk, dairy products, wool, lamb meat, etc.) from these animals [9]. Business owners can overcome this challenge provided that they ensure production outside the breeding season [9]. Regarding follicular dynamism, it is well-established that sheep ovaries are not completely stagnant but dynamic during anestrus. It has been suggested that there is continuous follicle-stimulating hormone (FSH) synthesis during anestrus and that there are fluctuations in follicular development in parallel with these FSH fluctuations [10,11,12]. Therefore, it was possible to introduce a production system in which pregnancies can be achieved after ovulary interventions based on the continuation of follicular activity during anestrus [13]. Off-season estrus induction methods are based on the use of progesterone and its analogs with equine chorionic gonadotropin (eCG) [14]. FSH and luteinizing hormone (LH) effects are utilized by applying eCG with the termination of P4 administration in sheep. eCG with dose regimens ranging from 250–750 IU is used for estrus synchronization in consideration of age, season, and breed [15]. Administered during or 24–48 h before exogenous P4 removal, prostaglandin F2α (PGF2α) provides luteolysis of corpus luteum, which may be present in some females during anestrus [16], and induces antral follicle development when co-administered with eCG [17]. Because human chorionic gonadotropin (hCG) has an effect similar to LH, it can stimulate oocyte maturation and ovulation, and therefore, in certain conditions, including luteal insufficiency, inadequate follicular maturation, and poor-quality oocytes and embryos, which may occur due to insufficient LH release, can be prevented by the use of hCG during the preovulatory phase [18,19,20]. 

It has been reported that resynchronization methods used to increase the success of estrus and ovulation in fixed-time artificial inseminations in cows provided a second insemination chance without wasting time in cows that did not become pregnant, thus increasing the pregnancy rate [21]. Although the effectiveness of exogenous progesterone supplementation on the corpus luteum and pregnancy in cows is well-known, the effectiveness of exogenous progesterone supplementation on the existing corpus luteum and newly formed pregnancy in sheep has not been fully elucidated [22,23]. No luteolytic agent is required after 12–14 days of progesterone use for inducing estrus and ovulation during the breeding season. In the scope of the resynchronization protocol, if no luteolytic agent is used during the second stimulation and that stimulation is performed only with progesterone support, then the second stimulation can be performed without the need for a pregnancy examination. Nevertheless, if a luteolytic agent is to be used, pregnancy must be confirmed at a very early stage [24]. Pregnancy losses in ewes are highest in the embryonic period, and there are a number of factors associated with the etiology of this problem. It has been reported that luteal dysfunction is the main problem in the occurrence of disruptions during pregnancy [25]. Pre-implantation losses are considered a serious issue in terms of maintaining effective reproduction in farm animals. Lack of fertilization accounts for only 5–10% of reproductive losses. Most of the losses are associated with embryonic deaths, which occur after Day 12 of pregnancy, prolonging the inter-estrous interval, and most ewes cannot mate again within the same season [26]. The main source of pre-implantation embryonal losses in ewes is associated with inadequate luteal function, and progesterone supplementation may prevent pregnancy losses in this context [27].

The present study aimed to demonstrate the effectiveness of the first resynchronization in ewes during anestrus. Accordingly, for the purposes of the present study, the second sponge was implanted in the resynchronization groups 14 days after the removal of the first and was retained in the vagina for 12 days, including the period when PGF2α was intensively released in order to prevent losses caused by pre-implantation luteal insufficiencies without a pregnancy diagnosis [26]. We aimed to reinforce implantation by means of a second sponge and, at the same time, to provide a chance for a second stimulation without delay when pregnancy did not occur. Furthermore, hCG was administered with an aim to ovulate or luteinize the follicles in order to develop follicles with high progesterone dominance, as seen in diestrus on the day of sponge administration. Therefore, better quality oocytes were expected, ensuring higher pregnancy rates.

## 2. Materials and Methods

This dissertation study was carried out in the Ulaş district of Sivas province, located at 39.316898 latitude and 36.994179 longitude. A total of 100 ewes of the Kangal breed were used as the study material. The sheep were 3 years old and biparous (weighing 55–72 kg), had conceived during the mating season, and were in the 50–75th postpartum days. The study also involved 3–5-year-old mature Kangal breed rams weighing 102–114 kg. The study was started during early anestrus (20 April). The rams were separated from the flock 25 days before the start of the study, and direct contact with the ewes was prevented. The ewes were fed only pasture feed and without extra feeding (flushing) before, during, and after the administrations. These points were considered in determining the materials for this study, and ewes that were 55–75 days postpartum that had terminated lactation were selected [16].

The dissertation study was commenced after decisions from the Selçuk University-Animal Experiments Local Ethics Committee No. 2018/21, dated 27 February 2018, and the Ministry of Agriculture and Forestry of the Republic of Turkey numbered 71037622-325.15-E 407736 and dated 8 February 2018, which accepted and approved the fact that all applications intended for the sheep and rams in the planned study were in line with animal rights and experimental ethics regulations. 

**Figure 1:**
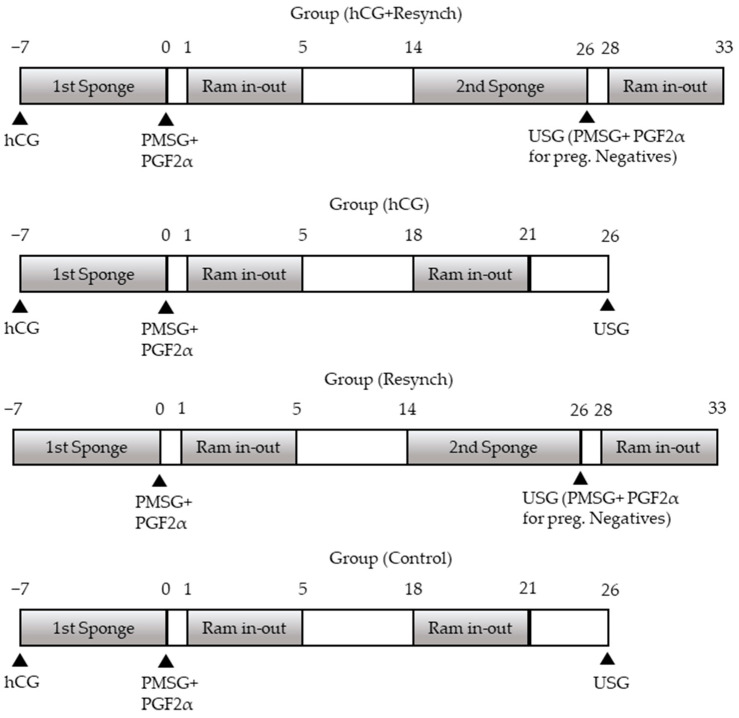
Treatment scheme of the groups during the study between day 7. and 33. PGF2α: prostaglandin F2α (131.5 µg cloprostenol, Estrumate); PMSG: equine chorionic gonadotropin (600 IU; Chronogest^®^ PMSG 6000); FGA: fluorogestone acetate (Sponge; 40 mg fluorogestone acetate, Chrono-gest), hCG: human chorionic gonadotropin (600IU Chorulon^®^).

The groups and scheduled protocols are shown in Figure 1. The groups were then divided into two further subgroups, namely the resynchronization subgroup (group (hCG+resynch) and group (resynch)) and the no resynchronization subgroup (group (hCG and group (control)). All the groups started with progesterone-containing sponge insertion on Day 7. The sponge was removed after 7 days (on Day 0), and 600 IU eCG (PMSG-Intervet^®^, MSD, Ankara, Türkiye) IM + 131.5 µg PGF2α (Estrumate^®^, MSD) was injected. The animals in group (hCG+resynch) and group (hCG) received 600 IU hCG (Chorulon^®^, MSD) IM injection at the time of sponge administration. Accordingly, four different groups were established, i.e., resynchronization + hCG administration (group (hCG+resynch); n:25), no resynchronization + hCG administration (group (hCG); n:25), resynchronization + no hCG administration (group (resynch); n:25), and no resynchronization + no hCG administration (group (control); n:25). Ram exposure was performed on Days 1–5 following the removal of the sponge. Mating was carried out by natural insemination. In the non-resynchronization groups, the second ram exposure took place on Days 18–21 (probably in their second cycles) following the first application, while all animals in the resynchronization groups were implanted with the second sponge on Day 14 (Day 0; sponge removal day), which was removed on Day 26 (after 12 days) when pregnancy examination was performed in all the groups. In the resynchronization groups, 600 IU eCG (PMSG-Intervet^®^, MSD) IM + 131.5 µg PGF2α (Estrumate^®^, MSD) IM injection was administered to sheep that tested negative for pregnancy, followed by the second exposure after 4 days. During the course of all these procedures, blood samples were collected for progesterone (P4) measurement. In all the groups, 30 days after the second ram exposure (in resynchronized groups), ultrasonographic pregnancy diagnosis was performed using the rectal method for the animals mated in the second ram exposure, while the second ultrasonographic pregnancy diagnosis was performed using the transabdominal method to determine whether there was embryonic death in the ewes that were pregnant after the first ram exposure on the same day. Third pregnancy examinations were performed to check whether the pregnancies resulting from the second ram exposure continued or not. Simultaneously with each treatment, 10 mL blood samples were taken from the vena jugularis for P4 analysis (during first sponge administration, at removal, 48 h after removal, second ram exposure [Day 18 for no resynchronization groups], second sponge administration, second sponge removal, 48 h after removal, and at all pregnancy examinations).

Pregnancy Examination was performed rectally in the supine position with a B-mode, linear-array 5.0–7.5 MHz rectal probe ultrasonography device (Mindray DP50/Vet/US) in order to determine early pregnancy and litter counts or transabdominally to detect embryonic and fetal losses that might have occurred during the subsequent days of pregnancy. The hairless area just above the breast, ventral to the right fasting pit, was preferred for probe placement for the purposes of transabdominal application. The dorso-caudal aspect of the breast was completely scanned based on whether pregnancy-related results could be found in that area. The examined animal was considered pregnant after the detection of the gestational sac, embryo/fetus, offspring membranes, fluids, heartbeat, and placentomes according to the period of pregnancy in the ultrasonic examination. Farm visits were made at specific intervals (monthly) to follow up on the pregnancy observed during the study procedures. Birth date and birth records were recorded at the daily visits to the facility 1 week before the anticipated day of birth.

Blood samples were kept at room temperature for half an hour and were placed in a refrigerated centrifuge at 3000 rpm for 5 min, and each of the serum samples were inserted into two 1 mL Eppendorf tubes and stored at −80 °C until the time of measurement. Progesterone levels were measured using the chemiluminescence microparticle immunoassay method using the ARCHITECT Progesterone Chemiluminescence (7K77) Abbott assay and the fully automated ARCHITECT-i2000SR (Abbott) instrument set at a progesterone analytical sensitivity of ≤0.1 ng/mL and a measurement range of 0.1–36.0 ng/mL progesterone. The intra-assay coefficient of variation ranged between 3.4–5.5% and 1.6–2.2% for low- and high-level progesterone concentrations, respectively. Analyses were validated for serum (in serum and blood collected in serum separator tubes) and plasma (with Na heparin, Li heparin, and K EDTA anticoagulants) samples. Validation was not performed with anticoagulants other than those mentioned above.

Descriptive statistics were calculated for all the variables measured for the purposes of the study. Prior to the significance tests, the quantitative data were tested for normality using the Shapiro–Wilk test and for homogeneity of variances using Levene’s test. Student’s *t*-test was used for intergroup comparisons for variables that met the assumptions of parametric testing, and the Mann–Whitney U test was used for variables that did not. For comparisons between more than two groups, a one-way analysis of variance was used for variables that met the parametric test assumptions, and the Kruskal–Wallis test was used for variables that did not. Tukey’s and Dunn’s tests were used as follow-up tests. Chi-squared analysis was used to determine the intergroup frequency distributions of categorical variables. Spearman’s correlation analysis was used to determine the correlation between two variables. A *p*-level of <0.05 was considered statistically significant. Statistical Package for the Social Sciences 25.0. (SPSS) (IBM Corp., released in 2017, IBM SPSS Statistics for Windows, Version 25.0., Armonk, NY, USA, IBM Corp.) software was used for all statistical analyses.

## 3. Results

Examination of the blood samples collected at the beginning of the treatment showed that all animals had sub-basal levels of progesterone (<1 ng/mL). None of the sponges intended for sexual stimulation, which constituted the basis of the study procedures, fell off spontaneously during the 7 days. While vaginitis was at an acceptable level when the first sponges were removed, vaginitis occurred in some sheep included in the resynchronization groups due to the prolonged use of vaginal sponges. Vaginitis with foul-smelling and dark-colored vaginal discharge was seen in a total of nine animals in the resynchronization groups: five sheep in group (hCG+resynch) and four sheep in group (hCG). Accordingly, the animals had increased vaginal reactions to the double use of vaginal sponges containing progesterone. Upon sponge removal and eCG and PGF2α injection, the time between the last administration and the onset of estrus was 30.4 ± 4.2 h after the first synchronization in all the groups, and the duration of estrus was 29.3 ± 3.8 h without any intergroup statistical differences, where the time between the last administration and the onset of estrus was 29.2 ± 4.4 h and the duration of estrus was 30.6 ± 2.5 h after the second stimulation in the resynchronization groups (group (hCG+resynch) and group (resynch)). 

Estrus rate at the first ram exposure was 76% (19/25), 88% (22/25), 96% (24/25), and 76% (19/25) in group (hCG+resynch), group (hCG), group (resynch), and group (control), respectively. There were no statistically significant intergroup differences in estrus rates (*p* = 0.785). Pregnancy rates at the first administration were 52% (13/25), 64% (16/25), 72% (18/25), and 60% (15/25), respectively, and there were no statistically significant intergroup differences (*p* = 0.539). Second estrous responses of 50% (6/12) and 85.7% (6/7) and pregnancy rates of 33.3% (4/12) and 28.5% (2/7), respectively, were observed in the resynchronization groups (group (hCG+resynch) and group (resynch)). There were no statistical differences between the resynchronization groups (group (hCG+resynch) and group (resynch)) in terms of the second estrus response (*p* = 0.173) and second pregnancy rates (*p* = 0.998) (Table 1).

The cumulative pregnancy rates were 68% (17/25), 64% (16/25), 80% (20/25), and 60% (15/25) in the group (hCG), group (resynch), and group (control) groups, respectively, and there were no statistically significant intergroup differences (*p* = 0.632) (Table 1).

Multiple pregnancy rates were 41.17% (7/17), 50% (8/16), 50% (10/20), and 53.33% (8/15) in group (hCG+resynch), group (hCG), group (resynch), and group (control) groups, respectively, and there were no statistically significant intergroup differences (Table 1).

**Figure 2:**
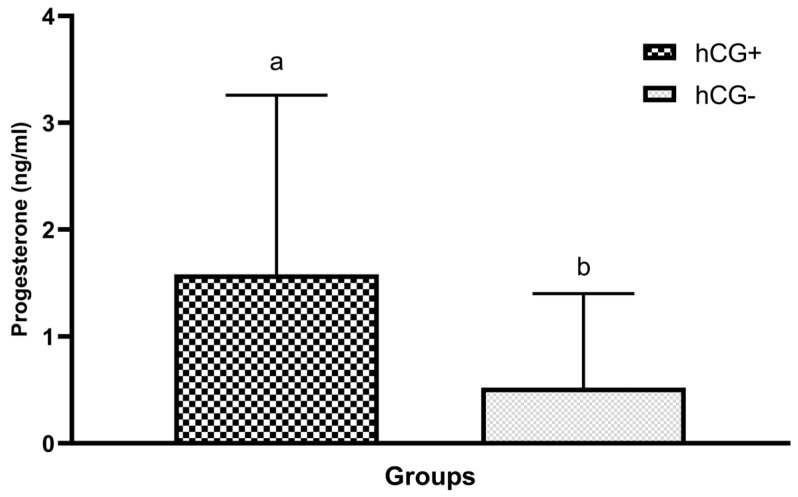
P4 values of groups treated and not treated with hCG on the day of sponge removal. a,b: Varied characters are statistically significantly different (*p* < 0.05).

Progesterone levels in blood samples collected at sponge removal 7 days after sponge administration were compared between all the study groups. The effect of hCG administration on progesterone levels was investigated after a comparison of groups treated and not treated with hCG. Furthermore, upon statistical analysis, the intergroup difference was considered statistically significant with a high positive correlation (*p* < 0.001) (Table 2 and Figure 2).

The efficacy of hCG was investigated after a comparison of the first estrus manifestation and pregnancy rates during the first estrus between the groups treated (group (hCG+resynch) and group (hCG)) and not treated with hCG (group (resynch) and group (control)) and no statistically significant intergroup differences were found in these two parameters (Table 3).

**Figure 3:**
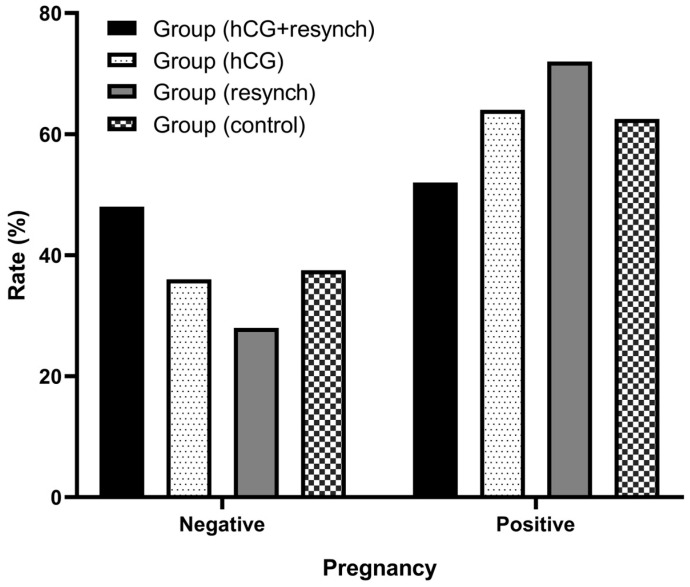
Graphical representation of pregnancy (first) status of the groups on Day 26.

The P4 levels and pregnancy rates at the first administration were compared between the groups based on data from Day 26. The intergroup difference for both parameters was statistically insignificant (Table 4 and Table 5 and Figure 3).

The relationship between P4 values on different days and multiple pregnancy statuses was also investigated. There was a positive correlation between P4 values on Day 26 and multiple pregnancy status (Table 6).

The distribution of multiple pregnancy rates between the groups and the effect of hCG administration and resynchronization on multiple pregnancy were investigated. The effect of these two treatments on multiple pregnancy was similar, and the difference was not statistically significant between the groups (Table 7).

In the beginning, the relationship between the live weight of the ewes and their pregnancy status was reviewed, and a very strong correlation was found. Accordingly, ram exposure was decreased when the live weight of the ewes exceeded the normal, and even if mating occurred, pregnancy rates were found to be lower in overweight ewes (Table 8).

## 4. Discussion

In the present study, reasonable rates of pregnancies were obtained in the first sexual stimulation, which did not include resynchronization. In particular, the high pregnancy rate at the first stimulation in the third group, which underwent resynchronization, prevented the exact determination of resynchronization efficiency.

Köse et al. (2013) investigated the effect of β-carotene or vitamin E + selenium injections on fertility during anestrus, and accordingly, 125 µg PGF2α (D-cloprostenol) and 400 IU eCG were injected 1 day prior to the removal of the sponge containing 20 mg flurogestone acetate that was applied for 10 days. While the study groups received vitamin supplements, the control groups only received hormonal stimulation. The pregnancy rate in the control group was 64.3% [28]. Therefore, similar pregnancy rates were achieved via short-term progesterone use and PGF2α–eCG injections on the day of sponge removal, reducing the workload for the facility and the stress for the ewes. Similarly, in a study with Awassi ewes during anestrus, vaginal sponges containing flurogestone acetate were applied to different groups for two consecutive short-terms, short-term, and long-term periods and 500 IU eCG was administered on the day the sponges were removed and pregnancy rates achieved were similar to those in the present study: 58.3%, 66.7%; and 58.3%, respectively [29]. Contrary to the above results, it was reported in another study that estrus formed more intensely in animals that were administered long-term progesterone (12 days) compared to those short-term progesterone (6 days) [30]. In the present study, short-term progesterone (7 days) was administered during the first synchronization, and long-term progesterone (12 days) was administered in the second phase in the resynchronization groups, and only the time between the last synchronization and estrus was investigated, which was similar in both groups. Although Ustuner et al. (2007) reported that estrus occurred more intensely with long-term P4 support compared to short-term support in their study, no such difference was observed in the present dissertation thesis study. It was considered that this might be associated with the injection of PGF2α at sponge removal in addition to eCG for the purposes of the dissertation. In addition, it was also hypothesized that the study by Ustuner et al. (2007) was carried out in the off-season period and that a lower dose (300 IU) of eCG was used [30].

In a different study carried out in anestrus, three different mating systematics were used for estrous induction using controlled internal drug release (CIDR) devices for 12 days, including natural ram exposure, estrous detection/insemination, and fixed-time insemination, with respective pregnancy rates of 55%, 29.4%, and 25% being achieved in the groups [31]. Since the exposures were carried out during early anestrus, higher pregnancy rates were achieved in the study groups using natural ram exposure. Therefore, it was concluded that short-term administration with low exposure to P4 could be preferred on the grounds that similar or higher pregnancy rates were achieved with short-term P4 administration.

P4 level was statistically significantly higher in groups that were administered hCG (*p* < 0.001). Nevertheless, there was no statistical difference between the hCG and no hCG groups in terms of estrus induction and pregnancy rates, which was attributed to the inability of the luteal structure to produce progesterone immediately after hCG administration. This is because a minimum of 3–4 days are required for the development of the corpus luteum after ovulation. The corpus luteum reaches its maximum size on Day 6 after ovulation when it produces the highest amount of P4 [32,33]. In the present study, since hCG was administered and a sponge was inserted, the sponge was removed, and eCG and luteolytic agent (PGF2α) were administered when the luteal structures that were likely to form after hCG administration had just reached the level of active P4 production. By this time, the follicles that would ovulate had completed their development under the progestative effect of the exogenous P4 source. In order to achieve the desired hCG efficacy in reproduction, it was concluded that hCG administration should be performed 4–5 days before sponge insertion, or if it is to be performed during sponge insertion, the sponge should be kept in the vagina for a prolonged length of time (12 days). Therefore, it was considered that hCG administration would ensure the development of follicles under a more intense progestative effect, similar to normal diestrus, and thus, estrus could be induced in more ewes, and higher pregnancy rates could be achieved.

Basal levels of progesterone and lower levels of circulating E2 (estrogen), LH, and FSH in ewes during anestrus reflect the characteristic hormone picture of the current reproductive period [32,34]. P4 levels were below basal levels in all the animals at the beginning of the present study. In the groups with no resynchronization (group (hCG) and group (control)), ovulation was induced during anestrus, and blood samples were collected on Day 18 (Day 0 when the sponges were removed) to determine whether the ewes had become cyclic in the following period and ram exposure was provided for 3 days. Most of the ewes had basal levels of P4 (<1 ng/mL), and only one ewe from each group (group (hCG), 1/9; group (control), 1/10) mated without a pregnancy outcome. In some ewes in anestrus, P4 secretion was above basal levels, and this is associated with the luteinization of antral follicles [10]. In the present study, the animals that did not show estrus induction and did not conceive upon the first administration in these groups did not show estrus in any of the subsequent cycles, and there was no case where P4 was higher than the basal level. In light of the above points, it was considered that Kangal ewes did not become cyclic and entered into deep anestrus again in cases where estrus and ovulation were achieved after the procedures carried out during anestrus and pregnancy did not occur after mating. The fact that the ewes that did not conceive, even in the groups that were predicted to have two consecutive ovulations, the first being at the time of hCG injection and the second one 7 days later when the sponge was removed (after estrus), did not show estrus in the following cycles is suggestive of the fact that the ewes returned to anestrus.

In a study conducted during the breeding season, a certain number of ewes were implanted with the first sponge containing medroxyprogesterone on Day −12 that was removed on Day 0, and after 12 days, ram exposure took place for 5 days. Half of the animals were exposed to the ram for 5 days from Day 14 to Day 20 after the second sponge was inserted and removed within 6 days without pregnancy examination, while the other half did not receive any treatment. Therefore, progesterone support was provided to those animals that achieved pregnancy during the first synchronization, while resynchronization was performed via a second stimulation without wasting time for those who did not become pregnant. The second stimulations were performed without a luteolytic agent because it was the breeding season. Statistical analyses were performed to assess the cumulative pregnancy rates, and the respective pregnancy rates for resynchronization and control groups were 62.3% and 67.3%, and there were no intergroup differences [24]. Regarding the present study, in which the first resynchronization was induced during the early anestrus period, six animals in the resynchronization group (hCG+resynch) showed second estrus, and four became pregnant, while six animals in the other resynchronization group (group (resynch)) showed second estrus and two became pregnant. In group (hCG+resynch), six offspring were achieved from four pregnancies upon resynchronization, while four offspring were achieved from two pregnancies in group (resynch). Cumulative pregnancy rates were compared between the hCG+ resynchronization group (group (hCG+resynch)) and the hCG + no resynchronization group (group (hCG)) and between the no hCG resynchronization group (group (resynch)) and the no hCG + no resynchronization group (group (control)), and there were no statistically significant differences. As a result, after an assessment covering all the groups in question, the intergroup difference in cumulative pregnancy rates was not statistically significant (*p* = 0.632). Although extra estrus and higher pregnancy rates were achieved, the lack of cumulative difference was attributed to the small number of animals in the groups.

Miranda et al. (2018) reported in their resynchronization study that exogenous progesterone support decreased the endogenous P4 levels without affecting the continuation of pregnancy [24]. In that study, the P4 level decreased similarly on Day 26 (*p* < 0.001) upon analysis of the endogenous P4 levels in the blood samples collected on Day 14 (Day 0: the day the sponge was removed) and 12 days after the insertion of second sponge (Day 26). In addition, vaginitis due to the second sponge implantation in some animals in the resynchronization groups and the P4 levels in the blood samples collected during the insertion of the second sponge (Day 14) were higher than the subluteal level, but there were no pregnancies detected during the pregnancy examination performed on Day 26; this suggests that severe vaginitis due to the second sponge implantation might have adversely affected pregnancy. Considering the fact that the study group was composed of Kangal breed sheep, which are suggested to have a high seasonal dependency, and that the study was carried out during anestrus, it was concluded that resynchronization could serve as an effective method after modification. It was considered that if the second P4 source was not administered intravaginally but through other routes (oral, ear implant, etc.), potential vaginitis could be prevented, ensuring higher rates of pregnancy. Cumulative pregnancy rates would be higher in the resynchronization groups, and higher pregnancy rates could be obtained during anestrus, provided that the aforementioned setback of resynchronization was prevented.

The efficacy of resynchronization on embryonic mortality could not be fully demonstrated since there was no embryonic death in the resynchronized and non-resynchronized groups. In order to determine the effect of resynchronization on embryonic mortality, further studies that perform resynchronization in periods with different embryonic mortality rates, applying different feeding regimes, body conditions, lactation statuses, and seasons are required.

In addition to the above points, upon assessment of the effect of the live weight of ewes before mating on the survival of embryos, we found that the survival rates of embryos and fetuses of underweight and overweight (obese) ewes were reduced [35]. In this dissertation study, upon analysis of body weights and pregnancy status prior to mating, it was found that excess body weight adversely affected conception rates. There was a statistically significant difference in the pregnancy rates between those with excess body weight and those with normal body weight (*p* < 0.001). It was considered that early embryonic death might have occurred in over-conditioned ewes.

The researchers found that the progesterone level was higher than that during diestrus (>5 ng/mL) for a period of 3–4 days following the administration of the P4-releasing device, yet the level decreased below the subluteal level (2 ng/mL) in the following days, and the continuous decrease in the plasma concentration of progesterone towards the end of the 11-day use period affected the LH release, oocyte development, and fertility [36]. At the same time, we found that the P4 concentration did not change after the implantation of two different intravaginal devices containing P4 (CIDR-G, DICO) in ovariectomized sheep. Similarly, in the present study, it was found that the progesterone concentration in blood samples collected at sponge removal had decreased below the subluteal level (2 ng/mL) and that the level of progesterone was similar to the values before sponge insertion.

Another study has reported a positive correlation between progesterone levels in blood samples collected as late as Day 120 of pregnancy and multiple pregnancies [37]. In the present anestrus study, the correlation between P4 levels in blood samples collected on different days and multiple pregnancy was investigated. On Day 26, there was a high positive correlation (*p* < 0.001) between progesterone levels in blood samples collected during the pregnancy examination and multiple pregnancies.

Sheep breeding is a more common source of income, especially in low-income and developing countries. Although the procedures for obtaining more lambs in these countries are expensive because of imported pharmaceuticals, they are not as valuable as obtaining extra lambs [38].

## 5. Conclusions

Thanks to resynchronization, time can be saved during off-season stimulation and the insemination of ewes for the second time in a short period, and pregnancy can be achieved. A reasonable number of pregnancies can be achieved via the second stimulation without losing time. If the side effects associated with the second progesterone exposure are eliminated, embryonic mortality can be prevented by starting the resynchronization on Days 12–14 of the cycle and applying the second P4 for a diestrus period (approximately 12 days). In addition, the injection of hCG during or before the administration of P4-containing sponges will induce ovulation or luteinization, leading to further P4 production. Therefore, it was concluded that the artificial diestrus activity provided by P4-containing sponges could be increased to a higher level by hCG administration, and new follicles would develop with more intense P4 activity. It is hereby suggested that follicles that develop with a more pronounced progestative effect may increase fertility.

## Figures and Tables

**Table 1:** Overall reproductive results of the study.

Parameters	Groups			
	(hCG+resynch) (*n*: 25)	(hCG) (*n*: 25)	(resynch) (*n*: 25)	(Control) (*n*: 25)
First estrus response	68% (19/25)	88% (22/25)	96% (24/25)	76% (19/25)
Pregnancy rate at first estrus	52% (13/25)	64% (16/25)	72% (18/25)	60% (15/25)
Second estrus response with resynchronization	50% (6/12)	-	85.71% (6/7)	-
Second estrus response with resynchronization	-	11.11 (1/9)	-	10 (1/10)
Second pregnancy rate with resynchronization	33.3% (4/12)	-	28.57% (2/7)	-
Second pregnancy rate with resynchronization	-	0	-	0
Cumulative pregnancy rate	68% (17/25)	64% (16/25)	80% (20/25)	60% (15/25)
Embryonic death	0	0	0	0
Number of offspring	*29*	25	31	25
Multiple pregnancy rate	41.17% (7/17)	50% (8/16)	50% (10/20)	53.33% (8/15)
**Productivity**	**29/17**	**25/16**	**31/20**	**25/15**

**Table 2:** Investigation of intergroup progesterone (P4) values on the day of sponge removal.

Group		Arith. Mean	Std. Error	Std. Dev.	Median	Min	Max	*p*
**hCG +**	sponge out day	1.58	0.24	1.68	0.76	0.18	7.93	<0.001
**hCG −**	sponge out day	0.52	0.12	0.88	0.28	0.11	5.56	

**Table 3:** Estrus and pregnancy rates in groups treated and not treated with hCG.

		Groups				
		hCG +		hCG −		
		*n*	%	*n*	%	*p*
Estrus (first)	No mating	9	56.30	7	43.80	0.785
	Mated	41	48.80	43	51.20	
Pregnancy status (first)	Null	21	56.80	16	43.20	0.451
	Pregnant	29	46.80	33	53.20	

**Table 4:** A comparison of P4 values of the groups on Day 26.

	Day 26 P4 Values ng/mL						
Group	Arithmetic Mean	Std. Error	Std. Deviation	Median	Minimum	Maximum	*p*
hCG+resynch	2.51	0.48	2.39	1.57	0.16	8.82	0.352
hCG	4.1	0.75	3.76	3.31	0.11	15.33	
resynch	3.81	0.52	2.62	4.02	0.18	10.36	
control	3.44	0.83	4.14	2.32	0.1	14.3	

**Table 5:** Intergroup comparison of pregnancy rates on Day 26.

	1. Pregnancy Status				
	Null		Pregnant		
Subgroup	*n*	%	*n*	%	*p*
hCG+resynch	12	48.00	13	52.00	0.539
hCG	9	36.00	16	64.00	
resynch	7	28.00	18	72.00	
control	9	37.50	15	62.50	

**Table 6:** The relationship between P4 level and multiple pregnancies on different days (unit of P4, values in ng/mL).

		Day 0	Day 2	Day 26	Number of Offsp.
Day 0	r	1			
	***p***				
Day 2	r	0.203	1		
	***p***	0.043			
Day 26	r	−0.035	−0.044	1	
	***p***	0.729	0.666		
Number of offspring	r	−0.108	−0.062	0.713	1
	***p***	0.285	0.538	<0.001	

**Table 7:** Distribution of multiple pregnancy between groups.

	Offspring Number						
Group	Arithmetic Mean	Std. Error	Std. Deviation	Median	Minimum	Maximum	*p*
hCG+resynch	1.16	0.25	1.25	1	0	5	0.68
hCG	1	0.18	0.91	1	0	3	
resynch	1.24	0.17	0.83	1	0	3	
control	1	0.2	1	1	0	3	

**Table 8:** Relationship between live weight and conception.

		Live Weight (kg)						
		Arith. Mean	Std. Error	Std. Deviation	Med	Min	Max	*p*
First pregnancy status	Null	64.5	1.05	6.36	68.7	51.6	72.5	<0.001
	Pregnant	57.7	0.41	3.23	57.9	51.8	66.8	
Second pregnancy status	Null	66.36	0.92	5.21	68.85	53.7	72.5	<0.001
	Pregnant	57.5	0.39	3.24	57.55	51.6	66.8	

## Data Availability

The data that support the findings of this study are available from the corresponding author Abdurrahman Takci upon reasonable request.
